# Longitudinal evaluation of the impact of school characteristics on changes in physical activity opportunities

**DOI:** 10.1371/journal.pone.0228716

**Published:** 2020-02-06

**Authors:** Janani Rajbhandari-Thapa, Justin Ingels, Kiran Thapa, Marsha Davis, Phaedra Corso

**Affiliations:** 1 Department of Health Policy and Management, College of Public Health, University of Georgia, Athens, GA, United States of America; 2 Department of Health Promotion and Behavior, College of Public Health, University of Georgia, Athens, GA, United States of America; 3 Kennesaw State University, Kennesaw, GA, United States of America; La Inmaculada Teacher Training Centre (University of Granada), SPAIN

## Abstract

**Background:**

Even as many states adopt physical activity policies to promote physical activity and prevent childhood obesity, little is known about differences in policy implementation based on school characteristics. We studied association of school characteristics and changes in physical activity opportunities at the school level during the implementation of a statewide physical activity policy in the state of Georgia.

**Methods:**

A web-based school survey was administered to elementary schools at two time points (before and during policy execution). Matched respondents (289 classroom teachers, 234 administrators) reported the frequency and duration of recess and integrated physical activity time. We used paired t-test to assess changes in physical activity opportunities and chi-square tests to assess the association of change in physical activity opportunities with school characteristics. We then constructed a multiple linear regression model following a change score method to identify school-level factors that predict the magnitude of change in physical activity opportunities.

**Results:**

There was an overall significant increase in total physical activity opportunities across time; however, schools with higher poverty showed a decrease in physical activity time by 5.3 minutes per day (95% CI: -9.2, -1.3). Further, the changes in physical activity time for schools in suburban Georgia were smaller (-5.7, 95% CI: -9.5, -1.9) compared to schools located in towns.

**Conclusions:**

The change in physical activity opportunities was not the same across schools and school characteristics predicted the magnitude of change. Additional efforts at the local level might be needed for equitable policy implementation based on schools’ geographical location and poverty level of the student population.

## Introduction

Childhood obesity in the United States (U.S.) remains an epidemic. According to a recent report, nearly 1 in 5 children aged 6 to 19 years in the U.S. are obese [1]. Despite some evidence of the overall stabilization of childhood obesity [2], recent studies suggest that childhood obesity is increasing [1–3] and more common among children in low socio-economic status [4]. Schools and communities with lower overall socio-economic status have been found to have more overweight children [5–6]. Childhood obesity and a sedentary lifestyle are also identified risk factors for an increased prevalence of hypertension, cardiovascular disease, and diabetes [7] with significantly high associated healthcare costs. The incremental lifetime medical cost of an obese child relative to a normal weight child who maintains this normal weight throughout adulthood is $19,000 per child [8].

A lack of physical activity, or a sedentary lifestyle, is an important contributing factor to the obesity epidemic [9–12]. Physical activity has many benefits among children, including improved fitness and body composition, better brain health, a higher likelihood of health when older, and a reduced risk of cardiovascular disease markers [13]. Integrated physical activity during class time has been shown to provide increased levels of moderate physical activity in children [14]. The U.S. Department of Health and Human Services recommends children get at least 60 minutes of moderate to vigorous physical activity daily for their health and well-being [13]. However, only one in four students meet this recommended level [15]. Schools are an important avenue to provide physical activity opportunities for two reasons: children spend half of their waking hours in school [16] and over 50 million students attend public schools [17]. The Institute of Medicine recommends that children receive half of their recommended physical activity time (30 minutes per day) while at school [18]. In response, school policies that increase the frequency, intensity, and duration of physical activity have been identified as vital in addressing the childhood obesity epidemic [19–20]. To promote policy impact on childhood obesity, an evidence base is needed to establish school-level factors that are associated with increased physical activity opportunities.

Statewide legislation targeting children in schools includes general health promotion, minimum nutritional standards for school meals, required reporting of student fitness, and required allotments for physical activity. According to one report, 44 states have mandated physical education for elementary schools, of which 22 require a fitness assessment [21]. In the state of Georgia where the prevalence of obesity (18.4%) among middle and high school students is eighth highest in the nation [22], the state legislature passed the Georgia Student Health and Physical Education (SHAPE) Act in 2009 to combat the epidemic. This Act requires each public school district to conduct an annual fitness assessment of all students enrolled in classes taught by certified physical education teachers (beginning in the 2011–2012 school year) [23]. This Act was also the impetus for a statewide initiative to increase physical activity among its children (referred to as the Ga Shape initiative). As a part of the Ga Shape initiative, public-private partnerships provides training and technical assistance to schools to aid in the implementation of statewide programs that promote physical activity. In addition, online and written resources are available for every elementary school in the state. A detailed timeline of state-level obesity efforts and additional details for the Ga Shape initiative have been published [24].

Previous studies that have evaluated the school training component of the Ga Shape initiative found the training sessions to be effective in increasing physical activity opportunities at schools in Georgia including before- and after-school activities, recess, integrated classroom time, and professional development opportunities for teachers [25–26]. Despite these positive findings, the results are limited by the cross-sectional design, which has been the most common design for studies that evaluate state and district physical activity-related policies [27–28]. These studies were also limited by the small sample of schools included in the analysis [29]. In this study, unlike previous studies, we utilize longitudinal data allowing an assessment of the school-level factors associated with changes in physical activity opportunities across time from a large sample of elementary schools across the state of Georgia. The purpose of this study was to first identify changes in physical activity time and then determine school-level factors that predicted higher physical activity time in schools. The results of these analyses will provide evidence to policymakers regarding potential school factors which may promote or prohibit increased physical activity time in schools. This is the first in a series of studies that will evaluate the process, reach, implementation, and impact of the Ga Shape initiative.

## Methods

### Data

The University of Georgia Human Subject Office determined the research as not human subjects’ research. The data for this study come from the Georgia Department of Public Health web-based school survey sent to all public elementary schools in the state of Georgia. A multidisciplinary team developed this online survey, adapting it from widely used and reliability-tested school physical activity tools [30]. The goal was to obtain one response each from a school administrator and a classroom teacher from each of six elementary grades (Kindergarten through 5^th^ grade) to assess physical activity opportunities as reported by administrators and grade level teachers [25]. The first wave of data collection occurred before the implementation of activities within the Ga Shape initiative (schools received the surveys between October 2013 and September 2014, hereafter referred to as the *first wave* of data). The second wave of data collection occurred roughly two years later (schools received the survey between September 2016 and May 2017, hereafter referred to as the *second wave* of data); 1087 and 624 schools participated in the first and second waves of data collection, respectively. A small portion of the data has been used in previous cross-sectional studies utilizing only the first wave [25–31]. No other studies to date have matched the full sample of schools across the two waves.

### Measures

We calculated total physical activity time using recess and integrated physical activity (in-class physical activity) time. We calculated recess and integrated physical activity time based upon the reported frequency (0, 1, 2, 3, 4, or 5 days per week) and duration of recess (<15, 15–19, 20–29, or ≥30 minutes per day), and duration of integrated physical activity time (0, 1–5, 6–10, 11–15, 16–20, 21–25, or >25 minutes per day). Total recess time for each grade was determined for both waves using either the classroom teachers’ and/or administrators’ responses. Integrated physical activity time was available by grade from classroom teachers only, while administrators provided a response meant to be representative of all grades at the school.

We used data on school-level characteristics from the Georgia Department of Education. These data included measures of school size, geographical setting, gender, race, and participation in free and reduced lunch (as an indicator of poverty). We divided schools into ‘majority male’ (schools having more than 50% male student population), ‘majority female’ (schools having more than 50% female population) and ‘gender equal’ (schools having an equal proportion of male and female students). Schools were classified by race composition into ‘majority white’ (schools having more than 70% white student population), ‘majority black’ (schools having more than 70% black student population), ‘white black’ (schools having more than 20% white and more than 20% black student population), and ‘multi’ (all other schools). Other school-level characteristics of interest were measures of poverty (‘complete’ for schools with 100% students receiving free/reduced lunch and ‘partial’ for schools with less than 100% students receiving free/reduced lunch), and school geographical location. Geographic location was categorized into ‘city’ (territory inside an urbanized area and inside a principal city-incorporated places with a large population of residents), ‘rural’ (Census-defined rural territory), ‘suburb’ (territory outside a principal city and inside an urbanized area), and ‘town’ (territory inside an urban cluster) based on Governor’s Office of Student Achievement [32].

We matched the responses from the two waves of data at the school level for each of the seven respondent categories (an administrator and six classroom teachers). At the administrators’ level, we matched responses from 234 schools. From each classroom teachers’ response, we averaged measures across grade and combined responses from classroom teachers to estimate a school-level response for 289 schools. In total, we had data in the two waves from 500 unique schools, which is close to 40% of all the elementary schools in the state of Ga. Grade-specific sizes vary from 56 to 173 matched responses from classroom teachers. S1 Fig provides details on the number of responses in the first and second waves and the number of matched responses.

### Analysis

The analysis of the change in physical activity from the first wave to the second wave of data collection followed three steps. *First*, we used paired t-tests to compare recess time by grade for responses from classroom teachers and administrators. We also compared integrated physical activity by grade using responses from classroom teachers. We compared changes in integrated physical activity from administrators’ responses at the school level, as responses were not available at the grade level. The goal of these exploratory analyses was to determine if there was a significant change in physical activity and check for consistency in these results as reported by the classroom teachers and administrators. *Second*, we conducted chi-square tests to analyze the association of school-level factors with changes in physical activity time from the first to the second wave of data collection. *Third*, we conducted multiple linear regression to identify school-level factors that predict magnitude of change following a change score method (the dependent variable is the change in physical activity from the first to the second wave of data) and a regressor variable method (the dependent variable is the physical activity during the second wave of data) [33]. The statistical significance is determined at a 5% significance level.

All the specifications in the linear regression controlled for the level of physical activity during the first wave of data collection. We conducted separate analyses for responses from administrators (234 schools) and classroom teachers (289 schools). The independent variables of interest were categorical variables representing the race, gender, and poverty compositions of each school; geographical location; and a continuous measure of school size (total number of students).

## Results

On average, we found significant positive changes in total (recess and integrated) physical activity time per day for grades 1 (4 minutes per day; 95% CI: 2.3, 6.5), 4 (6 minutes per day; 95% CI: 3.4, 7.9), and 5 (8 minutes per day; 95% CI: 4.0, 12.4) (Table 1).

**Table 1:** Mean recess, integrated and total physical activity (minutes per day) by grade level.

		Total			Recess time			Integrated time	
	N*	1^st^ wave	2^nd^ wave	N	1^st^ wave	2^nd^ wave	N	1^st^ wave	2^nd^ wave
Classroom teachers’ response									
Kindergarten	60	33	35	62	20	21	62	13	14
		p = 0.47			p = 0.67			p = 0.45	
Grade 1	155	29	33	169	18	20	157	11	13
		p<0.001			p = 0.03			p<0.001	
Grade 2	57	27	30	64	17	18	57	10	12
		p = 0.26			p = 0.41			p = 0.16	
Grade 3	48	29	31	56	18	18	48	11	13
		p = 0.22			p = 0.82			p = 0.04	
Grade 4	138	24	30	169	16	18	145	8	12
		p<0.001			p = 0.02			p<0.001	
Grade 5	50	22	30	62	14	17	51	8	13
		p<0.001			p = 0.04			p<0.001	
Administrator's response									
Kindergarten	171	25	29	194	20	21			
		P<0.001			p = 0.11				
Grade 1	171	24	28	194	19	20			
		P<0.001			p = 0.18				
Grade 2	170	24	28	195	19	20			
		P<0.001			p = 0.07		198	5	8
Grade 3	166	23	28	193	18	20	p<0.001		
		P<0.001			p = 0.05				
Grade 4	163	22	27	189	17	19			
		P<0.001			p<0.01				
Grade 5	159	22	27	185	17	19			
		P<0.001			p<0.01				

*N = Number of schools, N for recess and integrated time do not add up to N for total because of missing observations.

The proportion of schools with an increase in total physical activity time by at least 5 minutes per day varied by grade between 42% and 53% of schools while the proportion of schools with a decrease of at least 5 minutes varied by grade between 19% and 39% of schools (Table 2).

**Table 2:** Proportion of schools (by grade) with increase, decrease, or no change in total physical activity time by at least 5 minutes per day from 1^st^ to 2^nd^ wave of data collection.

	Kindergarten	Grade 1	Grade 2	Grade 3	Grade 4	Grade 5	p value
Classroom teacher’s response							
No. of schools	60	155	57	48	138	50	0.422
Increase	43%	50%	42%	46%	53%	64%	
No change	20%	22%	19%	25%	22%	14%	
Decrease	37%	28%	39%	29%	25%	22%	
Administrators’ response							
No. of schools	171	171	170	166	163	159	0.979
Increase	45%	43%	45%	48%	50%	50%	
No change	32%	34%	34%	33%	31%	31%	
Decrease	23%	23%	21%	20%	20%	19%	

The results on total physical activity time stratified by gender, race, poverty and geography suggest a significant relationship of change in physical activity time with geographical location (p-value 0.003, classroom teachers), race (p-value 0.001, administrators), and free and reduced lunch participation (p-value 0.012, administrators) (Table 3).

**Table 3:** Mean total physical activity in minutes per day (SD) by school characteristics.

	N	1^st^ wave	2^nd^ wave	Change	p value
**Classroom teacher’s response**					
Gender					
Majority male	200	26.9 (9.8)	32.1 (8.1)	5.2 (11.9)	0.989
Majority female	59	25.9 (9.2)	31.1 (7.1)	5.2 (9.7)	
Gender equal	5	26.3 (8.8)	32.2 (5.8)	5.9 (9.6)	
Race					
Majority White	45	29.7 (9.4)	33.7 (6.2)	4.0 (10.8)	0.137
Majority Black	47	20.0 (7.6)	28.2 (9.1)	8.2 (12.3)	
White/Black	94	28.3 (9.2)	32.1 (7.6)	3.8 (11.0)	
Multi	78	27.0 (9.7)	32.7 (7.5)	5.8 (11.4)	
Free/reduce lunch				
Complete	31	19.1 (7.9)	26.3 (6.5)	7.2 (10.3)	0.302
Partial	233	27.7 (9.4)	32.6 (7.7)	4.9 (11.5)	
Geography					
City	75	24.2 (9.3)	30.6 (8.3)	6.4 (13.2)	0.003
Rural	67	25.1 (10.0)	32.8 (7.2)	7.8 (11.6)	
Suburb	103	29.3 (9.4)	31.4 (7.7)	2.1 (9.6)	
Town	19	27.9 (8.3)	36.1 (7.5)	8.3 (7.9)	
**Administrators response**					
Gender					
Majority male	149	23.9 (9.1)	27.5 (8.1)	3.7 (11.2)	0.380
Majority female	37	23.2 (7.0)	29.7 (8.1)	6.4 (10.0)	
Gender equal	2	19.9 (16.4)	26.8 (8.1)	6.8 (8.3)	
Race					
Majority White	46	26.7 (6.3)	28.3 (8.4)	1.7 (9.0)	0.001
Majority Black	27	15.8 (8.0)	26.2 (7.9)	10.3 (10.4)	
White/Black	56	25.7 (9.3)	27.4 (8.3)	1.7 (10.5)	
Multi	59	23.1 (8.3)	29.0 (7.8)	5.8 (11.9)	
Free/reduce lunch					0.012
Complete	14	13.7 (10.1)	25.0 (7.7)	11.3 (13.0)	
Partial	174	24.5 (8.2)	28.2 (8.1)	3.7 (10.7)	
Geography					
City	37	20.1 (10.2)	28.2 (8.0)	8.1 (12.0)	0.086
Rural	56	24.1 (7.6)	26.8 (8.4)	2.7 (10.6)	
Suburb	77	24.2 (8.1)	28.2 (8.0)	4.1 (10.8)	
Town	18	27.7 (10.2)	29.4 (8.5)	1.7 (9.8)	

Matching schools based on classroom teachers and administrators’ response yielded 289 and 234 schools respectively. However, the N in the table does not add up to 289 for classroom teachers, and 234 for administrators due to missing data; 25 observations with missing data from classroom teachers and 46 observations with missing data from administrators.

Results from the regression analysis show a decrease in physical activity time by 5.3 minutes per day (p-value <0.01) for schools with all students receiving free and reduced lunch based on classroom teachers’ response. Results based on classroom teachers’ responses also suggest differences in the change in physical activity time by geographic region with smaller change in physical activity time for schools in suburban (-5.7, p-value <0.01) geographies compared to schools located in towns. The results also show diminishing marginal change in physical activity based on baseline levels of physical activity. A one minute per day higher physical activity opportunity in baseline was associated with a smaller increase in physical activity time by nearly one minute. (-0.9, p-value <0.01). We ran similar models using the physical activity time reported at the second wave as the dependent variable (rather than the change in physical activity time) with similar results. In these models, the coefficients on physical activity at baseline were not significant at the 5% level, while all other coefficients were the same as the models reported in Table 4.

**Table 4:** Beta coefficients from multiple linear regression with change in total physical activity time (1^st^ to 2^nd^ wave) regressed on school level factors.

School level factors	Classroom teachers	Administrators
	β (95% CI)	β (95% CI)
Total physical activity at 1^st^ wave	-0.912*** (-1.016, -0.808)	-0.882*** (-1.032, -0.733)
Gender equal		0.623
Majority female	-1.450	
	(-8.467, 5.567)	(-11.254, 12.499)
Majority male	-0.768	-1.705
	(-7.580, 6.043)	(-13.354, 9.945)
Majority black		
Majority white	2.798	2.003
	(-1.276, 6.871)	(-3.395, 7.402)
Multi race	2.272	1.965
	(-1.247, 5.790)	(-2.899, 6.830)
Majority white and black	0.768	-0.046
	(-2.722, 4.258)	(-4.974, 4.882)
Partial free and reduced lunch		
Complete free and reduced lunch	-5.254***	-2.414
	(-9.179, -1.329)	(-8.185, 3.356)
Town		
City	-3.478	0.632
	(-7.468, 0.512)	(-4.381, 5.645)
Rural	-3.943	-3.019
	(-7.888, 0.001)	(-7.659, 1.620)
Suburb	-5.709***	-1.733
	(-9.497, -1.921)	(-6.172, 2.706)
Total students	-0.001	0.001
	(-0.006, 0.003)	(-0.004, 0.007)
Constant	34.666***	26.058*** (13.272, 38.844)
	(26.138, 43.195)	
Observations	264	188

*** p<0.01

** p<0.05

## Discussion

The goal of this study was to evaluate the school-level factors that are associated with changes in physical activity time. Federal guidelines recommend that schools provide at least 30 minutes of physical activity opportunities to their students to help them attain the recommended level of at least 60 minutes per day [13]. Overall, the results in this study suggest that schools in the state of Georgia were increasing the physical activity opportunities offered to students during the time the Ga Shape initiative was in effect, up to an average of eight minutes per day. The average recess time varied by grade from 14 to 20 minutes per day in the first wave and increased to 17 to 21 minutes per day in the second wave. Similarly, the average total physical activity time varied by grade from 22 to 33 minutes per day in the first wave and increased to 30 to 35 minutes per day in the second wave. On average, during the study period the elementary schools surveyed in this study offered the recommended 30 minutes per day of total physical activity to their students, regardless of grade.

Provision of the recommended 30 minutes of physical activity opportunity to students through recess and integrated classroom time was not consistent across all schools. Further, schools varied in the change in physical activity time with decreased time in some schools. We were specifically interested in identifying elementary school characteristics that explained the magnitude of change in physical activity time. When all students in the school received free and reduced lunch, a proxy measure for poverty, the positive change in physical activity time per day was smaller by 5.3 minutes compared to schools where only some students received free and reduced lunch. Previous studies have also reported low levels of physical activity and/or high obesity among students in schools with lower overall socioeconomic status [34–35]. In addition, in our results, schools in suburban geographies reported a smaller positive change in physical activity per day by 5.7 minutes compared to schools in towns. This result follows previous findings on regional differences in self-reported physical activity. A study with 1687 boys and 1729 girls from 4^th^, 5^th^, and 6^th^ grade from urban, small cities, and rural Iowa showed rural-urban differences in physical activity [36]. Our results contradict older studies that have shown no differences between rural and urban schools in physical activity time [37]. Our study found an average of five minutes per day difference in the magnitude of total physical activity time change in poor and suburban schools. Five minutes per day of lost physical activity translates to 15 hours of physical activity time per student per year based on 180 days of school. The mean number of students per school in our sample was nearly 650. Fifteen hours less physical activity time per year for each student translates to a potential loss of roughly 9,750 hours of physical activity time and therefore a loss of the associated benefits of physical activity [13].

In this study, we also found that administrators reported more recess time, less integrated physical activity time, and less total physical activity time when compared to classroom teachers. This suggests that administrators may not be fully aware of the time devoted to integrated physical activity by classroom teachers as an effort to increase physical activity for their students. Withholding recess time to enforce school discipline might be one reason for lower recess time reported by classroom teachers [38]. Second, changes in physical activity time were larger for higher elementary grades while total physical activity time reported during the second wave was greater for the younger elementary grades. Older grades may have seen a greater increase in time as FitnessGram^©^ testing and reporting is required beginning in the fourth grade [23], focusing the need for more physical activity on these grades. However, on average recess and integrated physical activity time was smaller for the higher grades.

A qualitative study found shifting social and political climate, organization and mobilization of diverse partners behind a common agenda, and development of strategies to overcome impediments to the legislative process as factors that determine policy adoption [39].Equitable policy implementation might need additional efforts at the local level, based on geographical and socio-economic disparities within an existent state-level policy through an ongoing refinement of existing policies. A blanket program and policy might not be equally effective. A review article on preventive medicine has also called for development and refinement of policies on physical activity environmental influences that include recess time [40]. Our study adds to the existing evidence. A recent study has called for practitioners and researchers in public health and other fields that contribute to obesity prevention to consider an equity-oriented framework for obesity prevention to guide ways to give greater priority to equity issues when undertaking policy strategies [41]. For example, participation in Recess Enhancement Program developed to address barriers in physical activity has been found to be significantly associated with higher rates of vigorous physical activity among public elementary schools in New York City [42]. In Georgia, the state legislature recently passed legislation that encourages elementary schools in the state to include an average of 30 minutes per day of supervised unstructured activity time, preferably outdoors [43], however, the bill was vetoed by the Governor.

This study has some limitations. While this study is longitudinal, it is limited to data from two waves. However, we matched the two waves of data at the school and respondent-level, thus our analysis allowed for accurate measurements of change in the minutes of physical activity per day during the policy execution period. Study results are based on physical activity time reported by teachers and administrators instead of objective measures from students such as accelerometer data. However, in this study the focus is on physical activity opportunities provided to students, therefore teachers and administrators may more accurately assess this compared to students. Further, the changes in physical activity opportunities in this study are limited to recess and integrated classroom time. Some schools may have also provided additional opportunities in the form of before and after school programs that were not measured in this study.

### Conclusions

Overall, these results suggest positive changes in physical activity opportunities in elementary schools during the period of statewide policy implementation. The findings also identify geography and poverty to be school characteristics which significantly impact levels of change in physical activity opportunities. The study is based on elementary schools in the state of Georgia and the results are important for Georgia policymakers and to the public-private partners who support the implementation of the physical activity initiative. In addition, the results provide important information and inform other states considering the implementation of FitnessGram© or other similar activities designed to decrease childhood obesity by increasing physical activity levels at schools. This study is an important contribution to the school-based physical activity literature where there is a lack of rigorous evaluation on the school-level factors associated with changes in physical activity opportunities, particularly when statewide policies are in effect. Future studies that compare policy implementation across schools that differ in access to resources and the characteristics of the population they serve can further inform legislators as they develop targeted policies to promote physical activity opportunities in schools.

## Supporting information

S1 Fig This is the survey respondents during the two waves of data collection. (DOCX)
