# Supplementary material for: Longitudinal evaluation of the impact of school characteristics on changes in physical activity opportunities
Source: PLoS One. 2020 Feb 6;15(2):e0228716. doi: 10.1371/journal.pone.0228716 (PMC7004365; doi:10.1371/journal.pone.0228716)
Supplement: S1 Fig — (DOCX) [file pone.0228716.s001.docx]

Administrators:

445 schools

K-teachers:

323 schools

Grade 1 teachers:

325 schools

Grade 2 teachers:

324 schools

Grade 3 teachers:

336 schools

Grade 4 teachers:

325 schools

Grade 5 teachers:

319 schools

Administrators:

256 schools

K-teachers:

87 schools

Grade 1 teachers:

259 schools

Grade 2 teachers:

95 schools

Grade 3 teachers:

80 schools

Grade 4 teachers:

260 schools

Grade 5 teachers:

78 schools

Administrators:

234 schools

K-teachers:

64 schools

Grade 1 teachers:

173 schools

Grade 2 teachers:

65 schools

Grade 3 teachers:

56 schools

Grade 4 teachers:

178 schools

Grade 5 teachers:

63 schools

Grade K-5 teachers:

289 schools

1^st^ wave

2^nd^ wave

1^st^ wave

2^nd^ wave

1^st^ wave

2^nd^ wave

1^st^ wave

2^nd^ wave

1^st^ wave

2^nd^ wave

1^st^ wave

2^nd^ wave

1^st^ wave

2^nd^ wave

Matched datasets

S1 Figure: Survey respondents during the two waves of data collection
